# An Orbital Mystery: A Unique Case of an Obsolete Orbital Implant With a Review of Orbital Implant Materials

**DOI:** 10.7759/cureus.30215

**Published:** 2022-10-12

**Authors:** Catherine D Anderson-Quiñones, William I Evans, Madison C Perchik, Peter K Wojcik, Jacquelyn Laplant, Brian T Fowler

**Affiliations:** 1 Department of Ophthalmology, Hamilton Eye Institute, The University of Tennessee Health Science Center, Memphis, USA

**Keywords:** implant exchange, obsolete materials, biomaterials, ophthalmology, orbital implant

## Abstract

Orbital implant materials have evolved greatly over the past century and include but are not limited to metal, ceramic, polymer, silicone, and glass. Knowledge of historically used materials is clinically relevant to patient care as certain materials carry a greater risk of migration, extrusion, infection, and limitations for imaging modalities utilized to visualize adjacent structures.

We report an unusual case of an 80-year-old male who presented to our community hospital with seizure-like activity. CT imaging of the brain revealed several white matter and cortex lesions with the largest lesion measuring 2.5 × 2 × 1.9 cm. The patient had a history of enucleation with placement of an orbital implant following a penetrating injury to the left eye at four years of age. Hounsfield scale analysis was read by radiology as being most consistent with a thin metallic shell surrounding the orbital implant. The potential for metallic material was consistent with the implant’s age and time of placement. Few reviews on ocular implant materials from this period exist in the current medical literature. A single case report discussing a hollow metal orbital implant with similar-appearing imaging was identified. Due to concern for possible metal implant materials, the patient underwent implant exchange so MRI imaging could safely be performed. Intraoperatively, the implant was identified as a clear, hollow, non-metallic, non-porous polymer sphere. Following surgery, the patient was able to undergo appropriate neuroimaging with subsequent diagnostic biopsy.

Current literature reviewing CT or photographic imaging of ocular implant devices prior to the 1940s is limited. This case highlights the importance of detailing materials historically used in orbital implants, their effects on clinical decision-making, and the utility of Hounsfield scale values to identify a material’s radiodensity on CT imaging.

## Introduction

Materials used in making intraocular implants have evolved greatly over the past century and include metal, polymer, and glass. Understanding historically used materials can be clinically relevant to patient care as certain materials pose limitations for the utilization of imaging modalities to visualize adjacent structures.

This article was previously presented as an academic poster at the 2022 Women in Ophthalmology Summer Symposium.

## Case presentation

**Figure 1:**
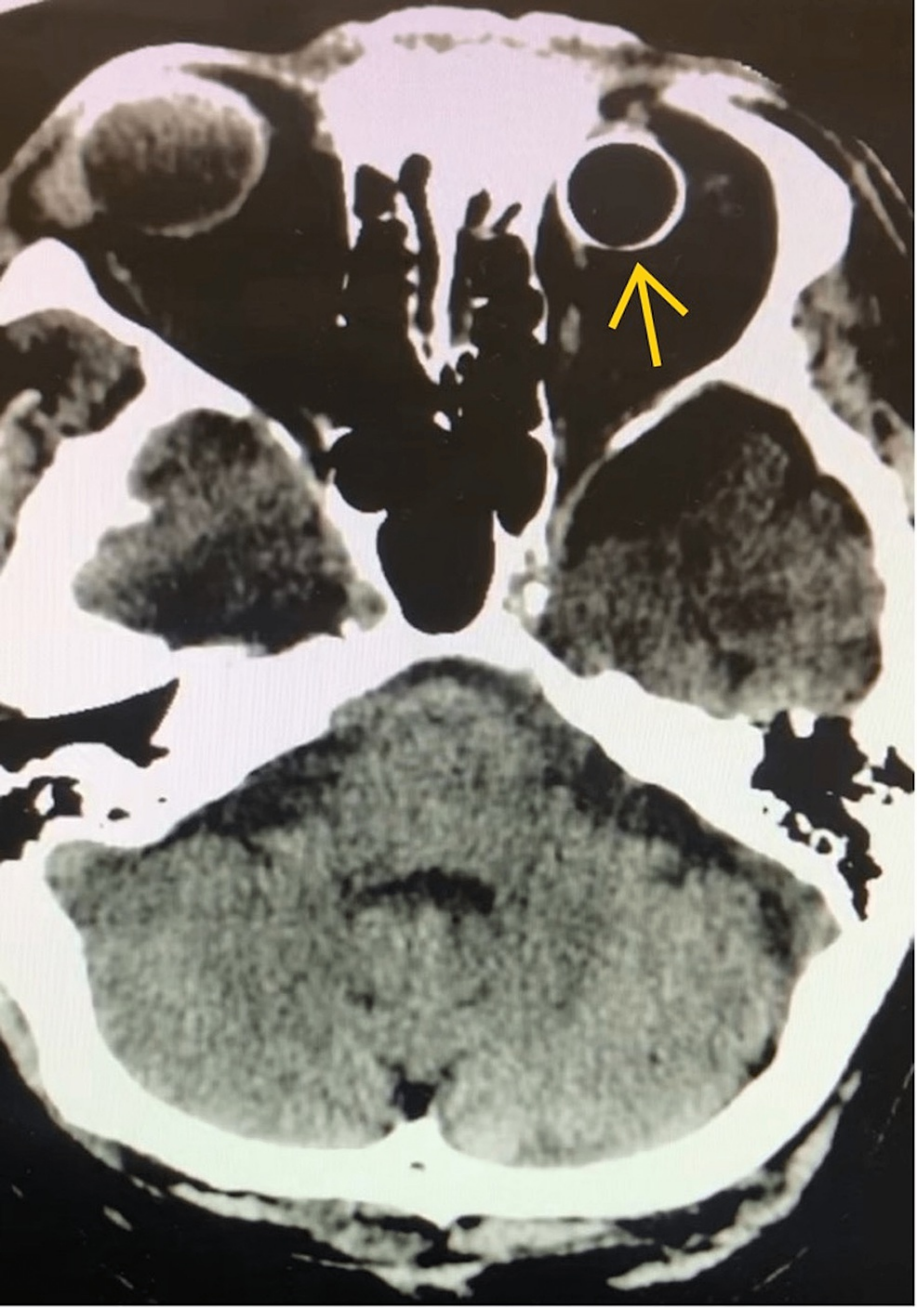
CT imaging of our patient, read by neuroradiology as having Hounsfield scale analysis consistent with a metal shell, showing similarities with imaging from a case report of a 1960s implant having a metal shell.

An 80-year-old male presented to the emergency department with fasciculations and seizure-like activity involving the right face and upper extremity. Presenting symptoms included twitching, which progressed to involve the right upper extremity, and dysarthric speech. The patient was given lorazepam and levetiracetam on admission and was admitted to the medicine service with consultations with neurology and neurosurgery. The patient’s past medical history included hypertension, hyperlipidemia, and chronic atrial fibrillation on rate control. His past surgical history included placement of a left orbital prosthesis following a penetrating injury as a child in the 1940s. Initial CT imaging without contrast was performed as part of hospital stroke protocol and revealed three areas of ill-defined subtle hyper-attenuation with a possible mass-like appearance involving the left corona radiata centrum semiovale and left frontal-parietal and parietal subcortical white matter and cortex. The largest lesion measured 2.5 × 2 × 1.9 cm. There was no associated edema or appreciable mass effect. Further characterization with MRI was recommended; however, the orbital implant was noted on the CT scan to have an unusual appearance (Figure 1).

The CT imaging was reviewed extensively with neuroradiology. The orbital implant was measured to be 1600 Hounsfield units, although radiology believed this to be artificially depressed due to the implant’s thin shape; the closest comparison was aluminum at 2000 Hounsfield units. Based on the appearance, age of the prosthetic, and measured radiodensity of the prosthesis on CT imaging, it was deemed to be a high probability that the implant consisted of a metallic shell. As such, MRI was not recommended due to the risk of thermal injury to the patient. These findings were discussed with the patient. Due to the need for lesion characterization, MRI-guided biopsy, and likely future surveillance MRIs, the patient agreed to proceed with left orbit exploration and implant exchange.

**Figure 2:**
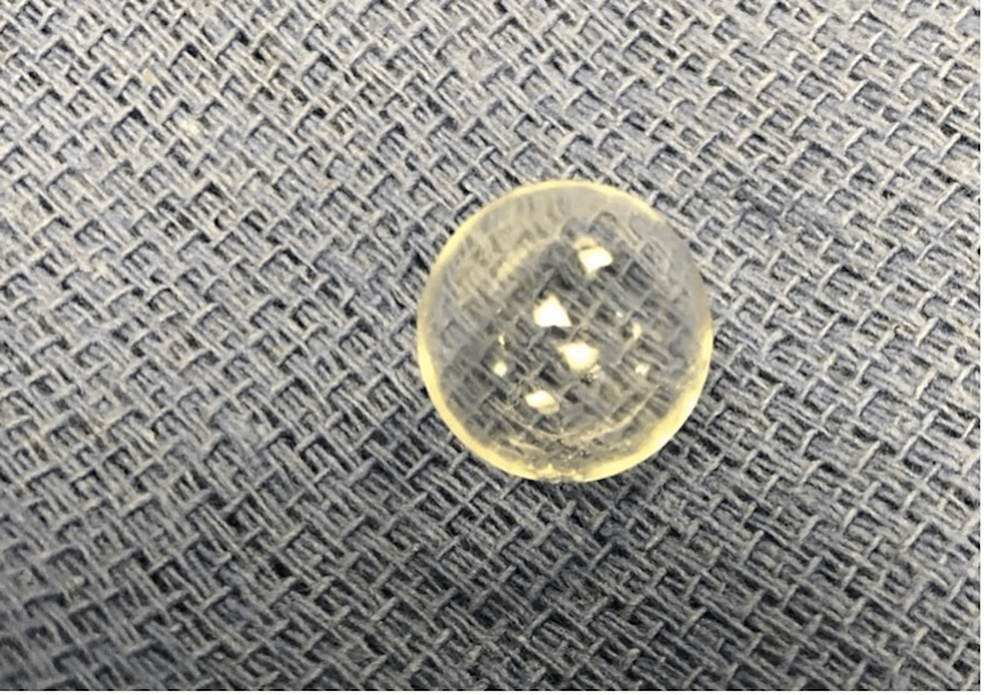
Obsolete early 1940s orbital implant after surgical removal, consistent with hollow, non-porous polymer material.

On exploration of the left orbit, the existing implant was identified to have migrated to the supranasal quadrant of the orbit. It was excised from the surrounding scar tissue and removed. The old implant was noted to be a clear, hollow, non-metallic, non-porous polymer sphere and was sent to pathology (Figure 2). A modern, MRI-compatible porous implant was placed into the orbit; the patient tolerated the surgical procedure without complication.

## Discussion

There are numerous medical indications for the removal of the eye, including non-reparable trauma to the globe, ocular malignancies, and pain in a non-functioning eye. After the removal of the globe contents, an orbital implant is placed into the anophthalmic socket. This orbital implant serves several purposes, including maintaining the space of the cavity, allowing for a symmetrical appearance of adnexal tissue, and providing structure for the fitting of a prosthesis to maximize the cosmetic outcome.

**Figure 3:**
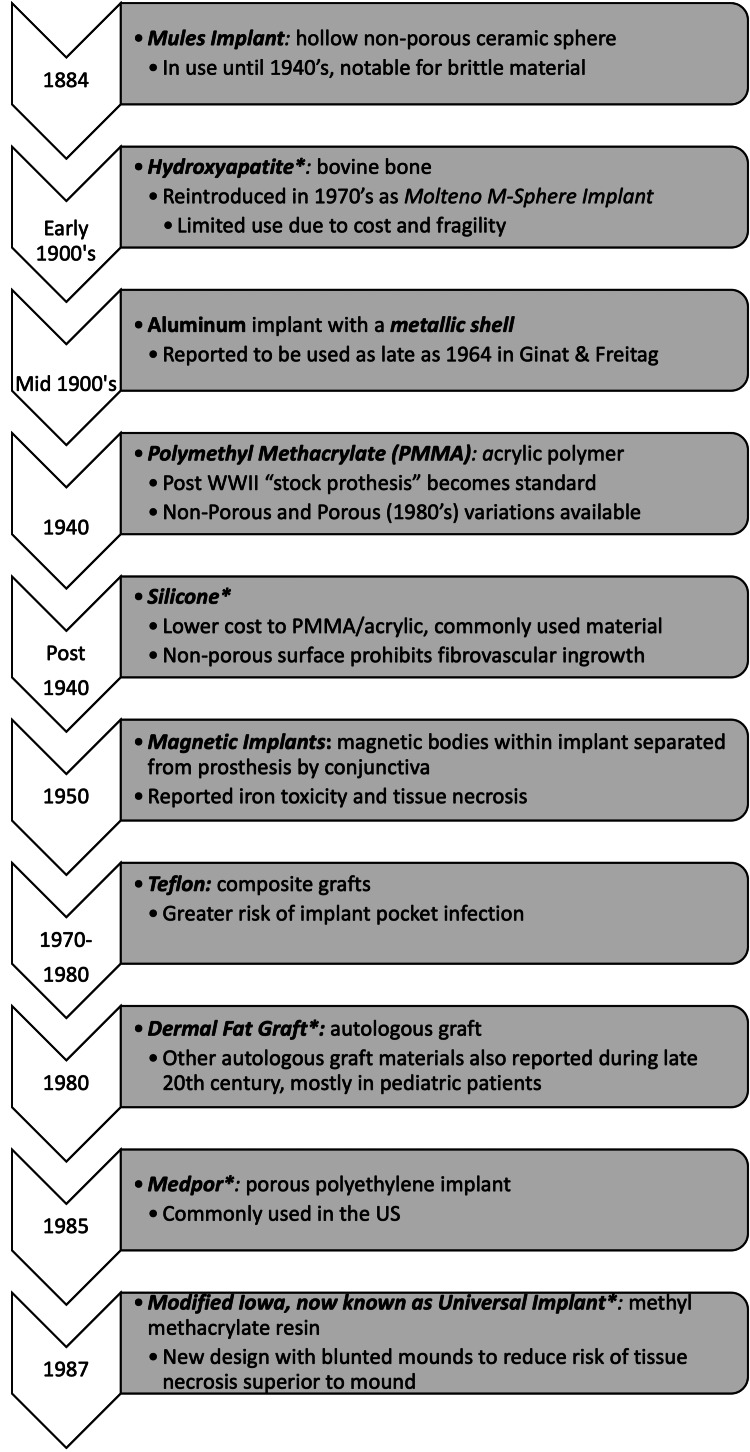
Timeline of materials used in orbital implants throughout the years *Implant material currently in use This figure is an original work complied by the authors to illustrate the prevalence of orbital implant materials throughout the years.

As a result, a thorough history of orbital implants in prior decades provides valuable information for the management of patients who have previously undergone enucleation. This is especially relevant for elderly patients who may have undergone enucleation with implant placement prior to the development of more modern orbital implants, as is the situation with our case. A timeline of implant development has been compiled to illustrate the evolution of materials used through the years (Figure 3).

Orbital implants were first developed in 1885 and were primarily composed of hollow ceramic or glass spheres. Advancements in orbital implants stalled until the 1940s when materials such as polymethacrylate (PMMA) and silicone began to be utilized. Both materials are smooth, non-porous, and non-integrated implants and are still in use today [1]. Innovation of polymer in implant materials developed due to limitations of availability for glass and ceramic materials during World War II [2]. Non-porous silicone implants also became available after the 1940s. These materials were lower in cost than PMMA/acrylic materials, but the non-porous surface inhibited vascular ingrowth [3].

Magnetic implants were also introduced in 1949 with the benefit that magnets both prevent extrusion and promote movement. One magnet was positioned at the posterior surface of the scleral shell prosthetic, while the other magnet with an opposite pole was positioned at the anterior of the orbital implant [4]. Additional movement could be achieved if more magnets were added in different planes. While magnetic implants did show low rates of extrusion, exposure and necrosis of tissue were not uncommon due to magnetic compression. These factors along with the inability of magnets to undergo MRI have resulted in these implants no longer being commonly used [2,4].

Teflon implants were introduced in the 1970s, but their use fell out of favor due to reports of complications, such as significantly increased rates of infection. Rates of infection were noted to be markedly increased with concurrent antral packing of Teflon sheets, and Teflon-based materials for orbital implantation are not currently available [5].

Porous implants were later developed in the early 1980s, which allowed for fibrovascular integration and muscular attachment [2]. Fibrovascularization benefits the patient by allowing for better anchoring of the implant to the host tissue, thus decreasing the risk of extrusion [6,7]. Muscular attachment improves the orbital implant’s ability to move. However, these changes were not met without risk, as there was still the danger of migration and extrusion along with postoperative infection.

Autologous materials, such as dermal fat grafts, have also been used in orbital implants with some success in younger patients. In theory, the autologous fat graft has the potential to vascularize and grow along with a child. Confounding issues secondary to radiation therapy often make the revascularization and success of the autologous graft implant challenging [3]. Autologous dermal fat grafts have also been used as primary or secondary orbital implants with success [8].

There is debate surrounding the best material for orbital implants. The currently used non-porous materials include PMMA and silicone, and the porous materials include alumina, polyethylene, and hydroxyapatite [9]. The current goals of implant development include counteracting the risks of orbital implantation by creating biomaterials that are antibacterial and angiogenic [2].

In this report, we document a unique case in which a patient with a remote history of left globe enucleation more than 70 years previously was found to have several intracranial lesions on CT imaging requiring subsequent MRI; however, it was uncertain whether the patient would be safe to undergo MRI as it was not known if the 1940s era orbital implant contained metal. This presented an interesting challenge on how best to identify whether the implant was metal or not, as the presence of a metallic implant would prohibit MRI necessary for lesion characterization, biopsy, and surveillance. Furthermore, the patient was in suboptimal health, and avoidance of unnecessary surgery was an important consideration.

The orbital implant appeared dissimilar to modern implants on CT imaging (Figure 1). Extensive discussion with neuroradiologists regarding the composition of the implant on imaging suggested that the implant contained metal as the radiodensity, measured in Hounsfield units, of the implant was most similar to aluminum. The Hounsfield scale is a quantitative method utilized by radiologists to delineate small contrast differences and decern a material’s composition based on density on CT imaging. Pixels, reflecting the electron density of the material in question, are assigned values in “Hounsfield units,” with water assigned a value of 0. All other densities are assigned values based on the following formula, with Mu as the CT attenuation coefficient. Metal, bone, and calcium result in scores of 1000 or more [10]. The implant was measured to be 1600 Hounsfield units, while the closest comparison, aluminum, is approximately 2000 Hounsfield units.

A thorough review of the literature was conducted with limited yield. A chapter from Ginat et al. highlighted an obsolete hollow metal spherical implant from 1964 that most closely resembled our patient’s implant on CT scan, again supporting the theory that this implant may contain metal [11]. However, surgical explantation confirmed a non-metallic, plastic polymer orbital implant. The thin nature of the implant’s shell likely impacted the Hounsfield unit measurement and resulted in an overestimation of the implant’s radiodensity. To our knowledge, this is the first documented case with imaging and photography of a polymer orbital implant from the 1940s.

## Conclusions

Orbital implant materials have evolved over the last several decades. Older patients may present with unusual-appearing implants, and a combination of history, imaging, measurement of radiodensity, and review of prior cases can assist in identifying what materials are present. The identification of which materials are present can play a significant role in clinical management, as was necessary in our case wherein a metallic implant would prevent the patient from undergoing MRI necessary for disease diagnosis and surveillance. Although our patient’s implant was not metal, it presented a similar picture. This case illuminates the potential clinical implications and limitations of using Hounsfield units to identify materials used in medical implant devices and the limited library of reference CT images of older orbital implants.

## References

[1] Schellini SA, El Dib R, Limongi RM, Mörschbächer R (2015). Anophthalmic socket: choice of orbital implants for reconstruction. Arq Bras Oftalmol.

[2] Baino F, Perero S, Ferraris S (2014). Biomaterials for orbital implants and ocular prostheses: overview and future prospects. Acta Biomater.

[3] Chen XY, Yang X, Fan XL (2021). The evolution of orbital implants and current breakthroughs in material design, selection, characterization, and clinical use. Front Bioeng Biotechnol.

[4] EL OH, LE OR (1956). A new magnetic orbital implant. AMA Arch Ophthalmol.

[5] Aronowitz JA, Freeman BS, Spira M (1986). Long-term stability of Teflon orbital implants. Plast Reconstr Surg.

[6] Salerno M, Reverberi A, Baino F (2018). Nanoscale topographical characterization of orbital implant materials. Materials (Basel).

[7] Anand R (2019). Commentary: analyzing the factors causing implant exposure in evisceration. Indian J Ophthalmol.

[8] Baum SH, Schmeling C, Pförtner R, Mohr C (2018). Autologous dermis - fat grafts as primary and secondary orbital transplants before rehabilitation with artificial eyes. J Craniomaxillofac Surg.

[9] Lukáts O, Bujtár P, Sándor GK, Barabás J (2012). Porous hydroxyapatite and aluminium-oxide ceramic orbital implant evaluation using CBCT scanning: a method for in vivo porous structure evaluation and monitoring. Int J Biomater.

[10] Kamalian S, Lev MH, Gupta R (2016). Computed tomography imaging and angiography - principles. Handb Clin Neurol.

[11] Ginat D, Moonis G, Freitag SK (2015). Imaging after oculoplastic surgery. Post-treatment imaging of the orbit.

